# Supplementary material for: Early Intervention and Lifelong Treatment with GLP1 Receptor Agonist Liraglutide in a Wolfram Syndrome Rat Model with an Emphasis on Visual Neurodegeneration, Sensorineural Hearing Loss and Diabetic Phenotype
Source: Cells. 2021 Nov 16;10(11):3193. doi: 10.3390/cells10113193 (PMC8623088; doi:10.3390/cells10113193)
Supplement: Supplementary file 1 [file cells-10-03193-s001.zip › cells-1431565-supplementary.pdf]

Supplementary Material

Title: Early intervention and lifelong treatment with GLP1 receptor agonist liraglutide in a Wolfram Syndrome rat model with an emphasis on visual neurodegeneration, sensorineural hearing loss and diabetic phenotype

Authors: Toomas Jagomäe *, Kadri Seppa, Riin Reimets, Marko Pastak, Mihkel Plaas, Miriam A. Hickey, Kaia Grete Kukker, Lieve Moons, Lies De Groef, Eero Vasar, Allen Kaasik, Anton Terasmaa, Mario Plaas *

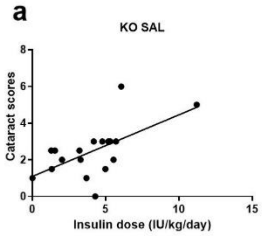

Goodness of Fit	
R squared	0.3963
Sy.x	1.120
Is slope significantly non-zero?	
F	11.16
DFn, DFd	1, 17
P value	0.0039
Deviation from zero?	Significant

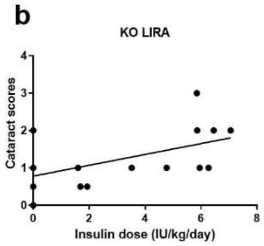

Goodness of Fit	
R squared	0.2713
Sy.x	0.6838
Is slope significantly non-zero?	
F	6.703
DFn, DFd	1, 18
P value	0.0185
Deviation from zero?	Significant

Figure S1. Correlation between insulin dose (IU/kg/day) and cataract score. (a) A positive correlation was observed between insulin dose (IU/kg/day) and cataract score in saline treated Wfs1 KO rats ($R^2 = 0.396$, $p < 0.01$) and in (b) liraglutide treated Wfs1 KO rats ($R^2 = 0.271$, $p < 0.05$).

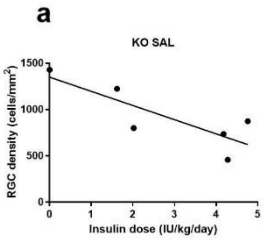

Goodness of Fit	
R squared	0.6757
Sy.x	223.4
Is slope significantly non-zero?	
F	8.336
DFn, DFd	1, 4
P value	0.0447
Deviation from zero?	Significant

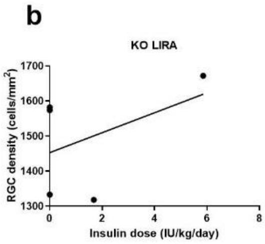

Goodness of Fit	
R squared	0.2030
Sy.x	165.2
Is slope significantly non-zero?	
F	0.7641
DFn, DFd	1, 3
P value	0.4464
Deviation from zero?	Not Significant

Figure S2. Correlation between insulin dose (IU/kg/day) and RGC density (cells/mm^2). (a) A positive correlation was observed in saline-treated Wfs1 KO rats between Insulin dose (IU/kg/day) and RGC density (cells/mm2) ($R^2 = 0.676$, $p < 0.05$). No significant correlation was found in (b) liraglutide-treated Wfs1 KO. The data were compared using linear regression analyses, n= 5-6.

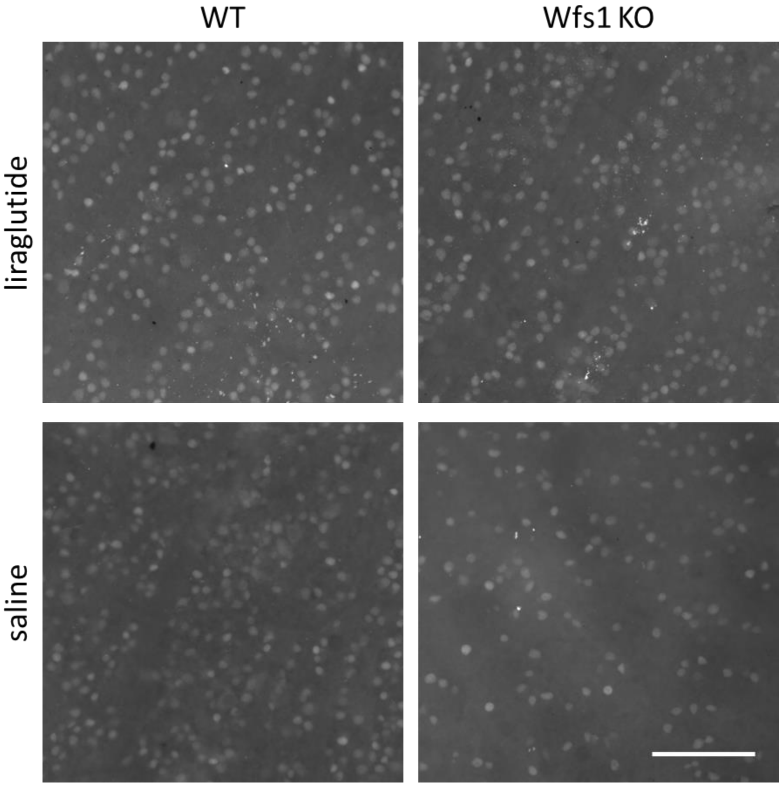

Figure S3. Representative images of Brn3a staining in retinal ganglion cells in every treatment group and genotype. Scalebar = 100 micron.
